# Effects of Weathering on Microplastic Dispersibility and Pollutant Uptake Capacity

**DOI:** 10.1021/acsenvironau.2c00036

**Published:** 2022-08-31

**Authors:** Ahmed Al Harraq, Philip J. Brahana, Olivia Arcemont, Donghui Zhang, Kalliat T. Valsaraj, Bhuvnesh Bharti

**Affiliations:** † Cain Department of Chemical Engineering, Louisiana State University, Baton Rouge, Louisiana 70803, United States; ‡ Department of Chemistry, Louisiana State University, Baton Rouge, Louisiana 70803, United States

**Keywords:** microplastics, accelerated weathering, surface chemistry, environmental fate, dispersibility, pollution, adsorption

## Abstract

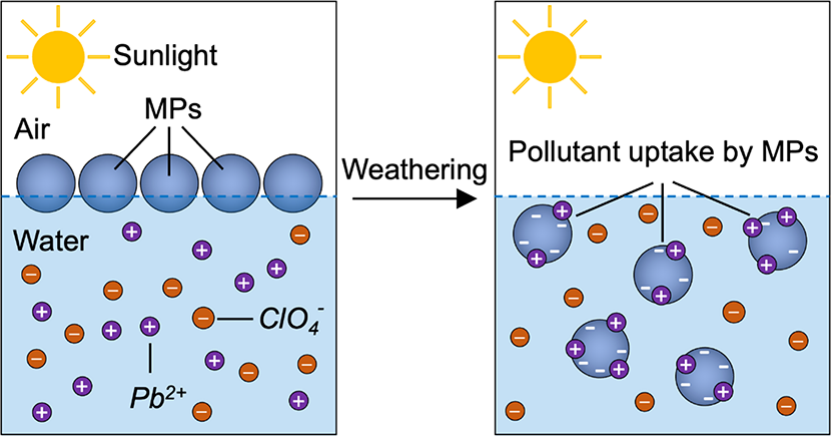

Microplastics are ubiquitous in the environment, leading to a new form of plastic pollution crisis, which has reached an alarming level worldwide. Micron and nanoscale plastics may get integrated into ecological cycles with detrimental effects on various ecosystems. Commodity plastics are widely considered to be chemically inert, and alterations in their surface properties due to environmental weathering are often overlooked. This lack of knowledge on the dynamic changes in the surface chemistry and properties of (micro)plastics has impeded their life-cycle analysis and prediction of their fate in the environment. Through simulated weathering experiments, we delineate the role of sunlight in modifying the physicochemical properties of microplastics. Within 10 days of accelerated weathering, microplastics become dramatically more dispersible in the water column and can more than double the surface uptake of common chemical pollutants, such as malachite green and lead ions. The study provides the basis for identifying the elusive link between the surface properties of microplastics and their fate in the environment.

## Introduction

Microplastics (MPs) are a growing threat to the environment, with reports indicating their widespread presence from urbanized areas to remote ecosystems.^1−4^ The ubiquitous nature of MPs poses a unique risk to both the environment^5^ and human health.^6^ MPs are readily available to marine biota by either direct ingestion or by trophic transfer through the consumption by polluted organisms.^7^ MPs are known to bioaccumulate in species that occupy lower-level trophic positions, such as oysters,^8^ mussels,^9^ and zooplankton,^10^ which enables these particles to eventually integrate into the human diet.^11^ This is particularly concerning given that MPs can act as a vector of transport for heavy metal and organic pollutants to enter the food web due to their ability to adsorb such chemicals.^12−14^

MPs have recently been found in human lungs,^15^ placentas,^16^ and blood.^17^ Their general toxicity and overall impact on health are difficult to assess due to variability in their chemical makeup, size, origins, and degradation pathways over time.^18,19^ MPs can be classified as primary when they are released into the environment as submillimeter-sized particles,^20^ for example, from commercial products in cosmetics, tires, and textiles.^21,22^ Conversely, secondary MPs are formed from the breakdown of larger plastic waste.^23^ Both primary and secondary MPs can be anticipated to undergo physical and chemical changes when released in the environment.^24,25^ However, there is a significant lack of understanding of the effect of parameters such as heat or sunlight irradiance on the physical properties of MPs that determine their long-term fate and interaction with the environment. Sustainability concerns regarding plastics are rendered more critical by the small size of MPs.^26^ We recently argued that this is due to the higher impact of surface properties at micro- and nanoscales and our insufficient knowledge of how these affect the environmental fate of particles.^27^

Determination of the dangers posed by MPs requires prediction of their long-term fate in the environment, which is a critical gap in current life cycle assessment methods.^28^ This highlights the need to relate the natural weathering that MPs experience in the environment with corresponding changes in surface chemistry that affect their key properties.^29^ In this article, we report the effect of sunlight-induced weathering of MPs on their dispersibility in water and their capacity to adsorb different classes of pollutants. The existing literature points to the photodegradation of plastics exposed to ultraviolet (UV) component of sunlight as an origin for the breakdown of macroplastics into small fragments.^30−33^ Nevertheless, it remains unclear how such weathering may affect the long-term physical state of both primary and secondary MPs. This is crucial because, with decreasing particle size, surface chemistry plays an increasingly important role in determining transport and settling dynamics,^34,35^ as well as the uptake of dissolved pollutant molecules.

## Results

We performed accelerated weathering experiments on model MPs. We selected polyethylene (PE) as our model MP because of its abundance in the environment, estimated to comprise >40% of plastic debris on marine surfaces.^36^ We employ commercially available PE microspheres (Cospheric LLC) of diameter ∼60 μm as model MPs to obtain general findings to correlate UV irradiance time with changes in dispersibility (Figure S1). The model PE spheres used were nearly neutrally buoyant in water with a mass density of 1.0 g cm^–3^, and the particles were labeled with a blue dye for improved visualization and analysis purposes. We further investigate the potential role of the particles as vectors for adsorption and transport of model environmental organic and inorganic pollutants, namely malachite green, 4-nitrophenol, lead, and perchlorate ions.

### Effect of Weathering on MPs

**Figure 1:**
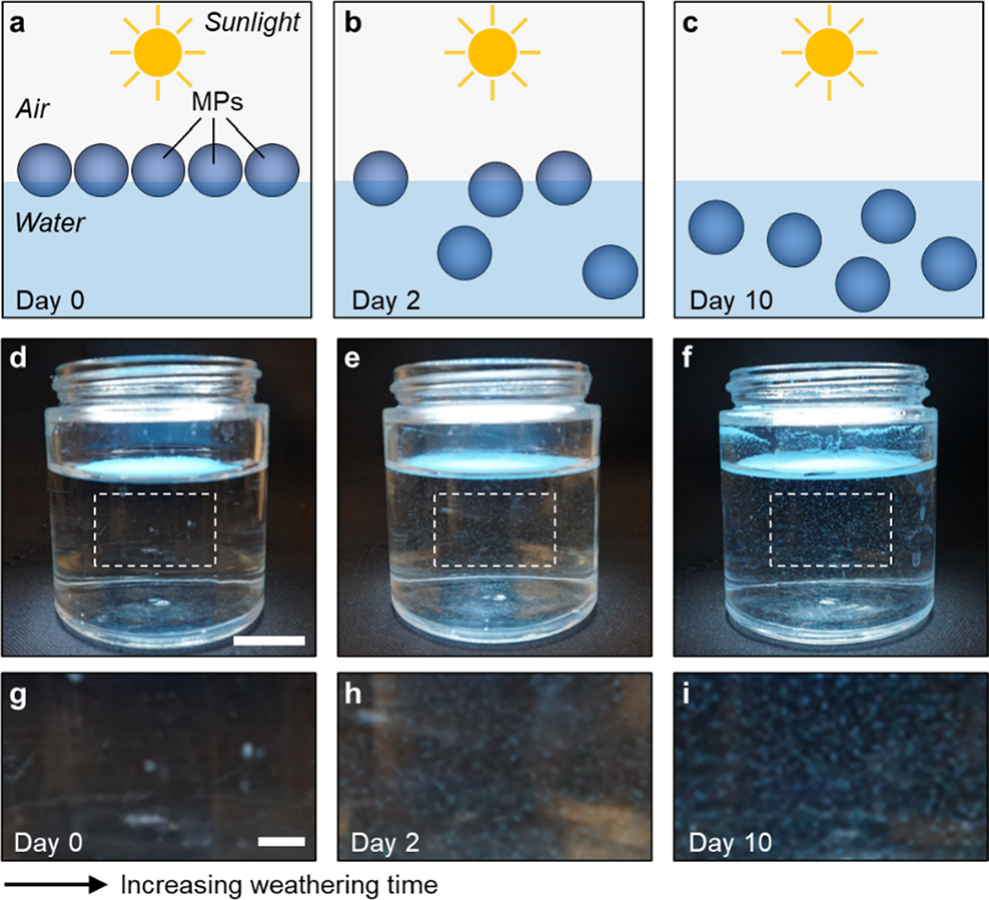
Increase in microplastic dispersibility by weathering. (a–c), Schematic, (d–f), photographs, and (g–i), zoomed-in highlights of the increase in dispersibility of polyethylene MPs (blue) in water after exposure to simulated sunlight, that is, weathering for 0 (left), 2 (middle), and 10 (right) days. The observed number of dispersed MPs is approximately 8, 140, and 237 particles cm^–2^, as reported in Figure 2a. Scale bars in d and g are 20 and 5 mm, respectively.

**Figure 2:**
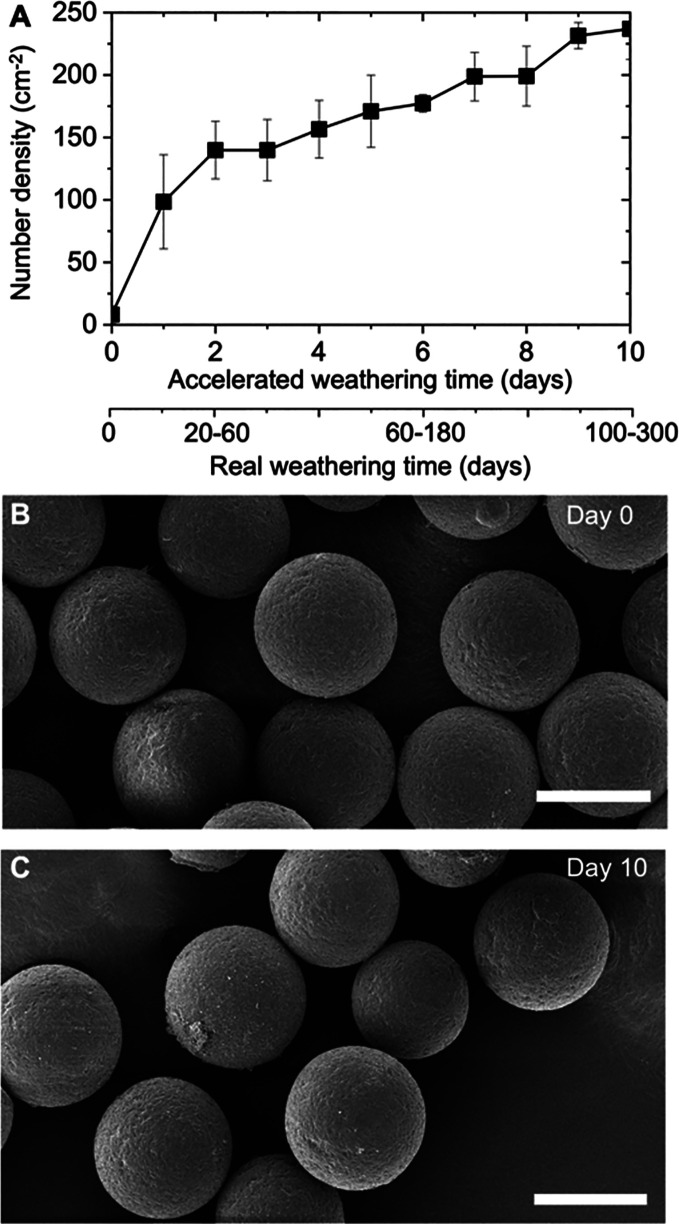
Effect of weathering on the dispersibility and morphology of microplastics. (a) Increase in the number density of MPs in the water as a function of weathering time estimated via image analysis. Dual x-axes are provided to indicate the accelerated weathering time inside the weathering chamber (top) and the estimated corresponding weathering time for a real outdoor environment (bottom). The number density of dispersed MPs is obtained from 2D images, as shown in Figure 1g–i; therefore, the units are cm^–2^. The bars are the standard error in the values obtained by at least three replicates of each measurement. Scanning electron microscope (SEM) images of microplastics (b) before and (c) after 10 days of accelerated weathering, indicating no significant change in the size and surface features. Scale bars: 50 μm.

Sunlight exposure to polymers, including polyolefins such as PE, causes their photooxidation, which can increase the wettability of the weathered plastics.^37^ We investigated the change in dispersibility of MPs in water over 10 days of accelerated weathering (Figure 1a–i). Cleaned glass containers were filled with 100 ml of deionized water and 100 mg of MPs that covered the air–water interface in approximately a single layer. These are placed in a weathering chamber where they are exposed to a xenon arc lamp equipped with a filter that limits UVB and UVC radiation. By limiting wavelengths in favor of UVA (∼340 nm) and setting the irradiance to 0.35 W m^–2^, we generate radiation which is comparable to natural sunlight according to ASTM D5071.^38^ Such irradiance is maintained constant inside the chamber, while the temperature is maintained around 63 °C via air cooling, which effectively accelerates the rate of photodegradation. In our experiments, weathering takes place at a rate accelerated by a factor that can range from approximately 10 to 30 (as estimated by TenCate Geosynthetics^39^). The exact factor depends on a host of parameters including latitude and altitude, but estimates can be obtained by comparing measurements of UV radiation at ground level with the irradiance measured inside the chamber. This means that 24 h of UV exposure inside the weathering chamber may correspond to between 10 and 30 days in real environments. For the sake of simplicity and to avoid inaccuracy, we report results as a function of the accelerated weathering time. We quantified the relative change in dispersibility of the particles in the water from UV exposure using image analysis^40^ (Figure S2). Initially, the MPs display high hydrophobicity resulting in virtually no particles leaving the water surface. We observed a sharp rise in dispersibility represented by the transport of interface-bound MPs toward the bulk water, which increases with weathering time (Figures 1,2a and S3). Note that the model MPs are neutrally buoyant, and thus the observed change in dispersibility can be caused by alterations in surface chemistry of the weathered particles. No change in the dispersibility was observed for MPs equilibrated in the dark, that is, in the absence of the UV light (Figure S4). The apparent increase in wettability indicates that due to sunlight exposure, even nominally hydrophobic MPs have the potential to sink in aqueous environments. Such rise in dispersibility happens before any observable change in the size and morphology of the particle takes place, as shown in Figure 2b,c. This suggests that the weathering process causes effects on microplastics properties, which occur sooner than their potential surface abrasion and breakdown into smaller pieces.^41^

### Origin and Consequence of Change in MP Wettability

**Figure 3:**
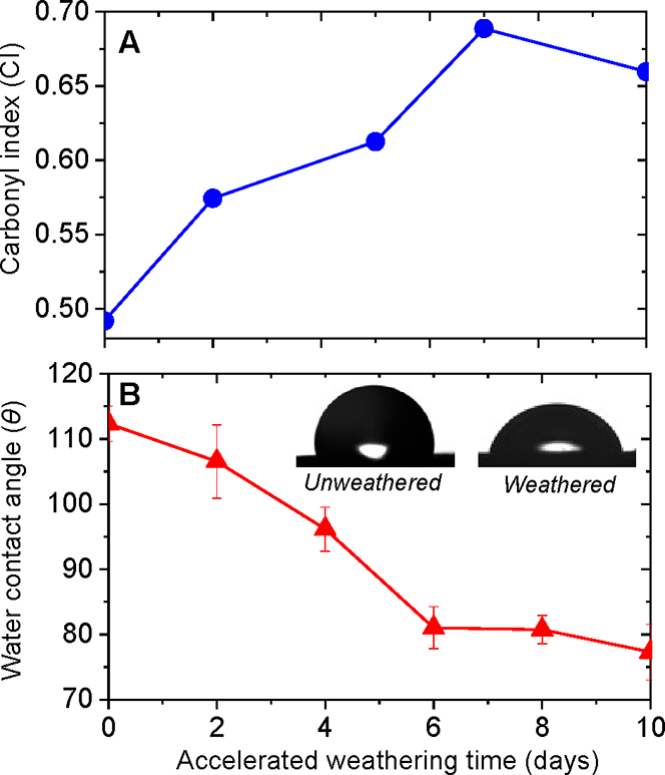
Physicochemical changes in surface properties of microplastics. (a) Carbonyl index increase measured via FTIR indicates an increase in carbonyl group formation with accelerated weathering time. (b) Decrease in the water contact angle measured as a function of accelerated weathering time. In the inset are images of sessile water droplets on unweathered and weathered PE, highlighting the transition from the hydrophobic to hydrophilic state. The bars are the standard error in the values obtained by at least three replicates of each measurement.

The reported change in dispersibility of the PE MPs is attributed to chemical transformation occurring due to exposure to UV light. Such weathering mechanism involves the photooxidation of the surface of MPs, which is normally quantified through the formation of carbonyl groups. We use Fourier-transform infrared spectroscopy (FTIR) to probe changes in the surface chemistry of MPs compacted into flat cm-sized pellets as a function of weathering time. The signature of carbonyl group formation in FTIR is the occurrence of a peak in the 1850–1650 cm^–1^ band. We monitored the change in transmission of the carbonyl band in comparison with the unchanging methylene scissoring peak observed in the 1500–1420 cm^–1^ band (Figure S5). The carbonyl index (CI), that is, the ratio between the specified areas under the carbonyl and methylene bands, provides a measure of photooxidation of polyolefins.^42^ We find that the CI of PE MPs increases within the first 10 days of accelerated weathering time (Figure 3a), highlighting the role of sunlight-induced chemical transformations in MPs. The initiation of the photooxidation process may be driven by the additives used in the manufacturing of the model MPs, and similar effects would be observed for commercial plastic materials where additives are generally present. To further corroborate this finding, we measured the water contact angle (θ) on the same pellets as a function of their accelerated weathering time. Using this semi-direct approach to measure wettability, we observed a decrease in the contact angle from a hydrophobic state, that is, θ > 90°, to a hydrophilic state of PE where θ < 90° (Figures 3b and S6). Similar changes take place on the surface of other plastics such as polypropylene (Figure S7).

### Surface Charge and Pollutant Adsorption on MPs

**Figure 4:**
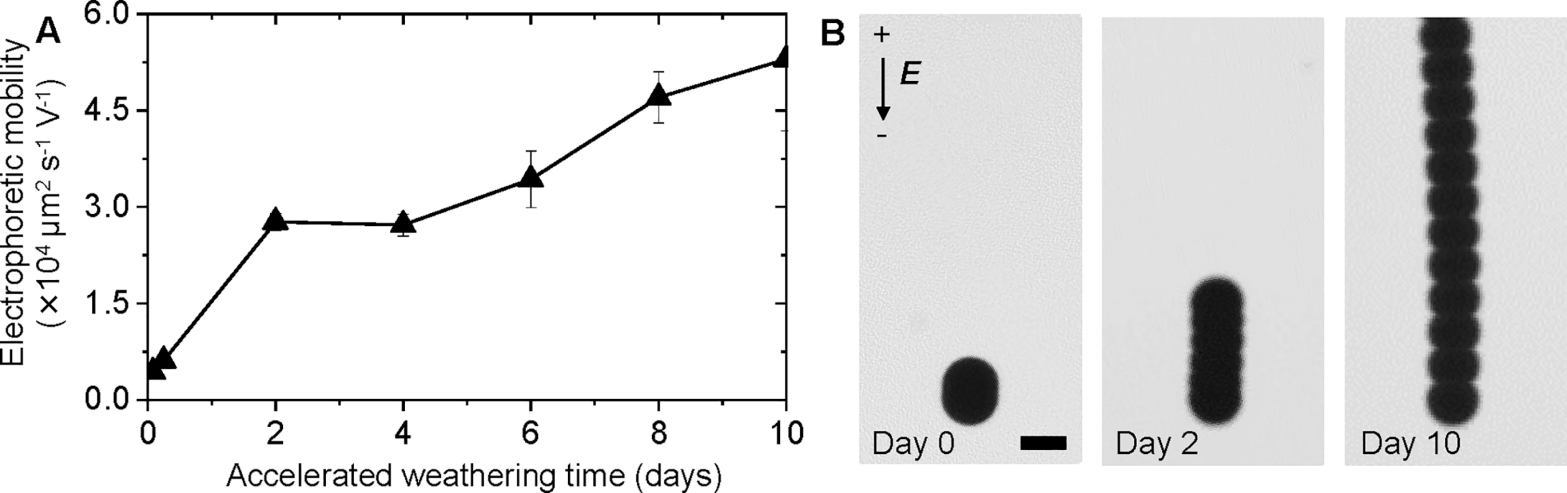
Electrophoretic evidence of an increase in the surface charge density. (A) Electrophoretic mobility of microplastics toward the anode increases with accelerated weathering time, highlighting the increase in the negative surface charge density. The error bars represent the standard deviation in at least four replicates of each measurement. (B) Superimposed microscopy images of PE microparticles moving for 5 s in the electric field (0.8 V mm^–1^) toward the positive electrode.

The weathering mechanism alters the surface charge density of MPs. We quantify the change by determining the electrophoretic mobility of MPs as a function of weathering time. The electrophoretic mobility μ_E_ is the terminal velocity *v* of a particle in an external electric field normalized to the applied field strength *E*, that is, μ_E_ = *v*/*E*. Here, *v* = (εζ/η)*E*, where ε is the dielectric constant of the medium, ζ is the zeta potential of the MP, and η is the viscosity of the medium. Thus, μ_E_ is linearly proportional to the zeta potential and dependent on the surface charge density of the particles.^43^ We perform our experiments in a custom-built setup composed of two coplanar electrodes fabricated via metal vapor deposition of a 100 nm gold film, with a 10 mm separation gap in which the aqueous MPs suspension was placed. The movement of the MPs under the influence of a direct current electric field is observed using an optical microscope in the brightfield mode. In our experiments, we find that the MPs move toward the anode, indicating that the net charge on the particles is negative. The magnitude of the electrophoretic mobility, and thus the surface charge density, increases with increasing weathering time (Figure 4a,b). Note that no change in the MP size was observed in our accelerated weathering experiments (Figure 2b,c); therefore, the observed change in electrophoretic mobility can only result from the changes in surface chemistry of the MPs. The negative charge on the MPs can be attributed to the dissociation of carboxylic acid groups formed at the surface of PE as a result of the photooxidation process. The mechanism of UV-induced photooxidation of macroscopic PE sheets can be found in previous studies.^44^ The increase in the electrophoretic mobility with weathering time highlights an increase in the density of the negative charges on the surface of the MPs. The introduction and increase in the negative charge on the MPs can affect their ecological impacts, specifically dispersion, transport, and adsorption properties.^27,45−47^

**Figure 5:**
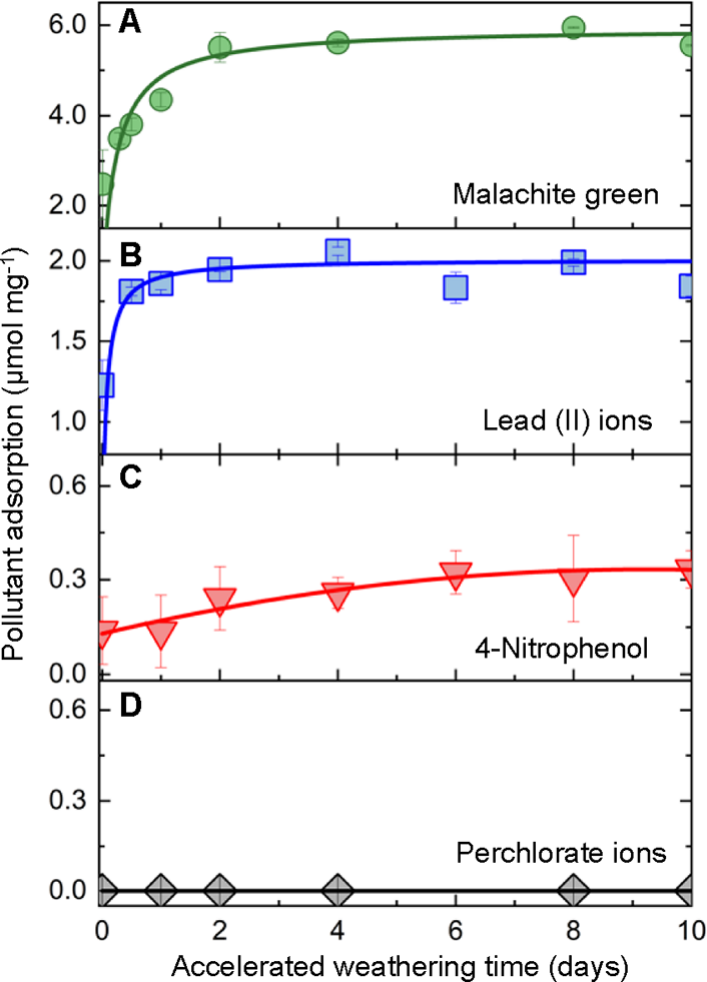
Changes in the pollutant adsorption capacity due to weathering. Adsorption of (a) malachite green, (b) lead (II) ions, (c) 4-nitrophenol, and (d) perchlorate ions as a function of accelerated weathering time. The symbols represent the experimental results as the concentration of pollutant per milligram of microplastics, and the lines are added for visual guidance and do not represent mathematical fits. The error bars represent the standard deviation in at least three replicates of each measurement.

The ability of MPs to adsorb pollutants dispersed in the surrounding medium can be substantially affected by changes in surface chemistry upon weathering.^48^ To probe the effect of weathering on pollutant uptake, we studied the adsorption of malachite green, lead ions (Pb^2+^), 4-nitrophenol, and perchlorate ions (ClO_4_^–^) onto MPs with an increasing degree of weathering. These pollutants were selected because of their potential toxicity and industrial relevance, such as in pesticide and fertilizer manufacturing and their persistence in aqueous environments. In addition, the selected chemicals display varying charges, which can help elucidate the adsorption mechanism and the effects induced by weathering on the surface charge density of MPs. The concentration of pollutant adsorbed onto MPs was determined by solvent depletion. In a typical experiment, 10 mg of MPs that were previously weathered for 24 h are equilibrated with a known concentration of the pollutant. The MPs with the adsorbed pollutant are separated from the aqueous dispersion by filtration. The amount of the nonadsorbed pollutant is determined either by spectrophotometry or using an ion-selective electrode, as detailed in the Methods section. The amount of the pollutant adsorbed on the MPs is estimated by subtracting the initial concentration of the pollutant in water and its equilibrium concentration in the filtrate. In our experiments, we find that the adsorption of malachite green doubles within 2 days of accelerated sunlight-induced weathering (Figure 5a), while the adsorption of Pb^2+^ increases by >50% during this period (Figure 5b). However, the amount of 4-nitrophenol adsorbed onto the MPs fluctuates around ∼0.2 μmol mg^–1^ (Figure 5c). In addition, ClO_4_^–^ does not showcase any detectable adsorption at any weathering stage (Figure 5d). The different trends observed can be attributed to the changes in electrostatic interactions driven by the weathering process. As the surface of MPs acquires negative charges, it attracts more Pb^2+^ and malachite green due to the positive charge on the quaternary amine group of these molecules. The adsorption of neutral molecules, such as 4-nitrophenol, is attributed mostly to the van der Waals forces and hydrogen bonding between 4-nitrophenol and the microplastic surface, which are largely unaffected by weathering. Analogously, negative ions, such as ClO_4_^–^, do not adsorb on the negatively charged surface of the MPs and may arguably be further repelled as the particles weather. These measurements indicate that in aqueous environments, the adsorption of pollutants onto MPs is dependent both on the surface chemistry of the particles and the adsorbing molecules. Such adsorption behavior will also be impacted by the pH, salinity, and temperature of the aquatic environment. Further studies are necessary to elucidate the complex relationships between the pollutant uptake capacity, weathering state, and environmental conditions.

In summary, we have shown that sunlight exposure drives a rapid transformation in physicochemical properties of MPs. Such changes in the dispersibility and adsorption capacity are critical in understanding the physical state of MPs but also in assessing their long-term transport and fate in the environment. MPs often assumed to be hydrophobic could become hydrophilic in the environment due to weathering that increases their dispersibility in water. As MPs become more dispersed throughout the water column, they may become more susceptible to vertical settling,^49^ deep-sea circulation,^50^ and also atmospheric transport via air-sea interactions.^2^ This finding contributes to our preliminary understanding of the reasons why large amounts of MPs are left unaccounted on the seafloor.^49,50^ Weathering also affects the pollutant uptake capacity of MPs, a factor of major concern and generally studied as a static property when it is in fact dynamic. Adsorption of toxic chemicals could magnify the negative consequences of microplastics accumulation and introduction into the food chain. Finally, increases in sunlight irradiance associated with factors such as ozone depletion and climate change^51^ render these findings more relevant to the formation and update of lifecycle assessments.^52^

## Methods

### Accelerated Weathering Experiments

Simulation of sunlight-induced weathering was done using a Xe-1 weathering chamber (Q-Labs) with a 340 nm wavelength filter and irradiance set at 0.35 W m^–2^, calibrated according to the ASTM D5071 standard. Each experiment was carried out by placing a glass container with 100 mg of microplastics in 100 mL of DI water. The number of particles was selected to ensure the formation of a single layer of microplastics at the water surface to avoid interparticle shading from the light source.

### Attenuated Total Reflection Fourier-Transform Infrared (ATR-FTIR) Spectroscopy

ATR FTIR was performed using a monolithic diamond crystal ATR accessory on a Bruker Alpha FTIR instrument. After blanking the instrument with air, measurements were taken by collecting 16 scans per spectrum at a 4 cm^–1^ resolution.

### Contact Angle Measurements

The contact angle between water and polyethylene was quantified using a Theta Attension optical tensiometer (Biolin Scientific). The measurements were done using a 2 μL sessile droplet of deionized water.

### Electrophoresis

Electrophoresis of microplastics was done using coplanar electrodes obtained by deposition of gold vapor on a microscope glass slide. The slide was soaked in NoChromix (Godax) solution for 12 h and then washed with DI water. An 8 mm wide paper mask is placed on the slide before coating with a 10 nm layer of chromium followed by a 100 nm layer of gold in a vacuum metal evaporator (Thermionics V@-90). The aqueous dispersion containing the microplastics is placed in the gap created by the paper mask between the electrodes. The electric field is applied and controlled by connecting the electrodes to a direct current power supply (BK Precision 1665).

### Optical Microscopy

Images of the microplastics and videos of electrophoretic mobility were recorded using a Leica DM6 microscope equipped with a Leica DFC9000 GTC digital camera. The objective used was a Leica ×5 air objective.

### Scanning Electron Microscopy

Scanning electron microscopy was done using a Quanta 3D DualBeam FEG FIB-SEM with an accelerating voltage of 5 kV. A droplet containing the microplastics was allowed to dry on carbon tape and was subsequently coated with a 5 nm layer of platinum to prevent charging.

### Pollutant Concentration Determination in the Filtrate

Detection of malachite green and 4-nitrophenol concentration in water was done using spectrophotometric methods. Lead and perchlorate ions concentration was determined using ion selective electrodes (Hanna HI4112 for lead, Oakton Cole-Parmer for perchlorate).

### Spectrophotometry

Pollutant solutions were prepared from reagent-grade malachite green (Acros Organics) and 4-nitrophenol (Tokyo Chemical Industry) in DI water. Adsorption experiments were done by dispersing 10 mg of MPs in either 70 μM malachite green solution or 20 μM 4-nitrophenol solution and allowing 24 h for equilibration. Before each measurement, particles were separated by filtering the solution through a 0.2 μm PTFE syringe filter. The concentration of unadsorbed molecules was determined using the spectrophotometric absorbance values at 618 nm for malachite green and 318 nm for 4-nitrophenol with a Nanodrop 2000. Absorbance values were converted to concentration following the Beer–Lambert law using experimentally obtained calibration curves (Figure S8). To estimate the amount of pollutant adsorbed on the surface of microplastics, the concentration of the unadsorbed pollutant was subtracted from the original concentration of the solution.

### Ion-Selective Electrode Detection

Pollutant solutions were prepared from reagent-grade lead nitrate (Sigma) and sodium perchlorate monohydrate (Fisher Chemical) dissolved in DI water. Adsorption experiments were done by dispersing 10 mg of MPs in a 150 μM lead nitrate solution or 180 μM sodium perchlorate solution and allowing 24 h for equilibration. The amount of unadsorbed lead and perchlorate ions were detected using the HANNA HI4112 lead ion-selective electrode and the Oakton by Cole-Parmer perchlorate selective electrode, respectively, which were calibrated daily. The amount of pollutant adsorbed on MPs was estimated by subtracting the detected value from the known original concentration.

## Supporting Information Available

MP size distribution, dispersibility quantification using image analysis, reference dispersity change in the absence of UV light, FTIR spectra and water contact angle of MPS at various accelerated weathering times, contact angle change in polypropylene due to weathering, and calibration curves for determining unknown concentrations of malachite green and 4-nitrophenol (PDF)

## References

[ref1] Woodward J.; Li J.; Rothwell J.; Hurley R. Acute Riverine Microplastic Contamination Due to Avoidable Releases of Untreated Wastewater. Nat. Sustain. 2021, 4, 793–802. 10.1038/s41893-021-00718-2.

[ref2] Allen S.; Allen D.; Phoenix V. R.; Le Roux G.; Durántez Jiménez P.; Simonneau A.; Binet S.; Galop D. Atmospheric Transport and Deposition of Microplastics in a Remote Mountain Catchment. Nat. Geosci. 2019, 12, 339–344. 10.1038/s41561-019-0335-5.

[ref3] Waller C. L.; Griffiths H. J.; Waluda C. M.; Thorpe S. E.; Loaiza I.; Moreno B.; Pacherres C. O.; Hughes K. A. Microplastics in the Antarctic Marine System: An Emerging Area of Research. Sci. Total Environ. 2017, 598, 220–227. 10.1016/j.scitotenv.2017.03.283. 28441600

[ref4] Andrady A. L. Microplastics in the Marine Environment. Mar. Pollut. Bull. 2011, 62, 1596–1605. 10.1016/j.marpolbul.2011.05.030. 21742351

[ref5] Zhou J.; Wen Y.; Marshall M. R.; Zhao J.; Gui H.; Yang Y.; Zeng Z.; Jones D. L.; Zang H. Microplastics as an Emerging Threat to Plant and Soil Health in Agroecosystems. Sci. Total Environ. 2021, 787, 147444 10.1016/j.scitotenv.2021.147444.

[ref6] Rahman A.; Sarkar A.; Yadav O. P.; Achari G.; Slobodnik J. Potential Human Health Risks Due to Environmental Exposure to Nano- and Microplastics and Knowledge Gaps: A Scoping Review. Sci. Total Environ. 2021, 757, 143872 10.1016/j.scitotenv.2020.143872. 33310568

[ref7] Nelms S. E.; Galloway T. S.; Godley B. J.; Jarvis D. S.; Lindeque P. K. Investigating Microplastic Trophic Transfer in Marine Top Predators. Environ. Pollut. 2018, 238, 999–1007. 10.1016/j.envpol.2018.02.016. 29477242

[ref8] Cole M.; Galloway T. S. Ingestion of Nanoplastics and Microplastics by Pacific Oyster Larvae. Environ. Sci. Technol. 2015, 49, 14625–14632. 10.1021/acs.est.5b04099. 26580574

[ref9] von Moos N.; Burkhardt-Holm P.; Köhler A. Uptake and Effects of Microplastics on Cells and Tissue of the Blue Mussel Mytilus Edulis L. after an Experimental Exposure. Environ. Sci. Technol. 2012, 46, 11327–11335. 10.1021/es302332w. 22963286

[ref10] Cole M.; Lindeque P.; Fileman E.; Halsband C.; Goodhead R.; Moger J.; Galloway T. S. Microplastic Ingestion by Zooplankton. Environ. Sci. Technol. 2013, 47, 6646–6655. 10.1021/es400663f. 23692270

[ref11] Cox K. D.; Covernton G. A.; Davies H. L.; Dower J. F.; Juanes F.; Dudas S. E. Human Consumption of Microplastics. Environ. Sci. Technol. 2019, 53, 7068–7074. 10.1021/acs.est.9b01517. 31184127

[ref12] Godoy V.; Blázquez G.; Calero M.; Quesada L.; Martín-Lara M. A. The Potential of Microplastics as Carriers of Metals. Environ. Pollut. 2019, 255, 113363 10.1016/j.envpol.2019.113363. 31614247

[ref13] Fang S.; Yu W.; Li C.; Liu Y.; Qiu J.; Kong F. Adsorption Behavior of Three Triazole Fungicides on Polystyrene Microplastics. Sci. Total Environ. 2019, 691, 1119–1126. 10.1016/j.scitotenv.2019.07.176. 31466193

[ref14] Bakir A.; Rowland S. J.; Thompson R. C. Transport of Persistent Organic Pollutants by Microplastics in Estuarine Conditions. Estuar. Coast. Shelf Sci. 2014, 140, 14–21. 10.1016/j.ecss.2014.01.004.

[ref15] Jenner L. C.; Rotchell J. M.; Bennett R. T.; Cowen M.; Tentzeris V.; Sadofsky L. R. Detection of Microplastics in Human Lung Tissue Using ΜFTIR Spectroscopy. Sci. Total Environ. 2022, 831, 154907 10.1016/j.scitotenv.2022.154907. 35364151

[ref16] Ragusa A.; Svelato A.; Santacroce C.; Catalano P.; Notarstefano V.; Carnevali O.; Papa F.; Rongioletti M. C. A.; Baiocco F.; Draghi S.; D’Amore E.; Rinaldo D.; Matta M.; Giorgini E. Plasticenta: First Evidence of Microplastics in Human Placenta. Environ. Int. 2021, 146, 106274 10.1016/j.envint.2020.106274. 33395930

[ref17] Leslie H. A.; van Velzen M. J. M.; Brandsma S. H.; Vethaak A. D.; Garcia-Vallejo J. J.; Lamoree M. H. Discovery and Quantification of Plastic Particle Pollution in Human Blood. Environ. Int. 2022, 163, 107199 10.1016/j.envint.2022.107199. 35367073

[ref18] Fleury J.-B.; Baulin V. A. Microplastics Destabilize Lipid Membranes by Mechanical Stretching. Proc. Natl. Acad. Sci. 2021, 118, e2104610118 10.1073/pnas.2104610118. 34326264 PMC8346836

[ref19] Andrady A. L. The Plastic in Microplastics: A Review. Mar. Pollut. Bull. 2017, 119, 12–22. 10.1016/j.marpolbul.2017.01.082. 28449819

[ref20] Al Harraq A.; Bharti B. Microplastics through the Lens of Colloid Science. ACS Environ. Au 2021, 1, 3–10. 10.1021/acsenvironau.1c00016. PMC10125150 37101760

[ref21] van Wezel A.; Caris I.; Kools S. A. E. Release of Primary Microplastics from Consumer Products to Wastewater in the Netherlands. Environ. Toxicol. Chem. 2016, 35, 1627–1631. 10.1002/etc.3316. 26627661

[ref22] Zitko V.; Hanlon M. Another Source of Pollution by Plastics: Skin Cleaners with Plastic Scrubbers. Mar. Pollut. Bull. 1991, 22, 41–42. 10.1016/0025-326x(91)90444-w.

[ref23] Sipe J. M.; Bossa N.; Berger W.; von Windheim N.; Gall K.; Wiesner M. R. From Bottle to Microplastics: Can We Estimate How Our Plastic Products Are Breaking Down?. Sci. Total Environ. 2022, 814, 152460 10.1016/j.scitotenv.2021.152460. 34973311

[ref24] Guo X.; Wang J. The Chemical Behaviors of Microplastics in Marine Environment: A Review. Mar. Pollut. Bull. 2019, 142, 1–14. 10.1016/j.marpolbul.2019.03.019. 31232281

[ref25] Leppänen I.; Lappalainen T.; Lohtander T.; Jonkergouw C.; Arola S.; Tammelin T. Capturing Colloidal Nano- and Microplastics with Plant-Based Nanocellulose Networks. Nat. Commun. 2022, 13, 1814 10.1038/s41467-022-29446-7. 35383163 PMC8983699

[ref26] Gigault J.; El Hadri H.; Nguyen B.; Grassl B.; Rowenczyk L.; Tufenkji N.; Feng S.; Wiesner M. Nanoplastics Are Neither Microplastics nor Engineered Nanoparticles. Nat. Nanotechnol. 2021, 16, 501–507. 10.1038/s41565-021-00886-4. 33927364

[ref27] Al Harraq A.; Bharti B. Microplastics through the Lens of Colloid Science. ACS Environ. Au 2022, 2, 3–10. 10.1021/acsenvironau.1c00016. PMC10125150 37101760

[ref28] Gontard N.; David G.; Guilbert A.; Sohn J. Recognizing the Long-Term Impacts of Plastic Particles for Preventing Distortion in Decision-Making. Nat. Sustain. 2022, 5, 472–478. 10.1038/s41893-022-00863-2.

[ref29] Steensgaard I. M.; Syberg K.; Rist S.; Hartmann N. B.; Boldrin A.; Hansen S. F. From Macro- to Microplastics - Analysis of EU Regulation along the Life Cycle of Plastic Bags. Environ. Pollut. 2017, 224, 289–299. 10.1016/j.envpol.2017.02.007. 28222979

[ref30] Liu Z.; Zhu Y.; Lv S.; Shi Y.; Dong S.; Yan D.; Zhu X.; Peng R.; Keller A. A.; Huang Y. Quantifying the Dynamics of Polystyrene Microplastics UV-Aging Process. Environ. Sci. Technol. Lett. 2022, 9, 50–56. 10.1021/acs.estlett.1c00888.

[ref31] Andrady A. L.; Lavender Law K.; Donohue J.; Koongolla B. Accelerated Degradation of Low-Density Polyethylene in Air and in Sea Water. Sci. Total Environ. 2022, 811, 151368 10.1016/j.scitotenv.2021.151368. 34732340

[ref32] Liu P.; Li H.; Wu J.; Wu X.; Shi Y.; Yang Z.; Huang K.; Guo X.; Gao S. Polystyrene Microplastics Accelerated Photodegradation of Co-Existed Polypropylene via Photosensitization of Polymer Itself and Released Organic Compounds. Water Res. 2022, 214, 118209 10.1016/j.watres.2022.118209. 35219184

[ref33] Wu X.; Liu P.; Wang H.; Huang H.; Shi Y.; Yang C.; Gao S. Photo Aging of Polypropylene Microplastics in Estuary Water and Coastal Seawater: Important Role of Chlorine Ion. Water Res. 2021, 202, 117396 10.1016/j.watres.2021.117396. 34246992

[ref34] Guo Y.; Lou J.; Cho J. K.; Tilton N.; Chun J.; Um W.; Yin X.; Neeves K. B.; Wu N. Transport of Colloidal Particles in Microscopic Porous Medium Analogues with Surface Charge Heterogeneity: Experiments and the Fundamental Role of Single-Bead Deposition. Environ. Sci. Technol. 2020, 54, 13651–13660. 10.1021/acs.est.0c03225. 33079526

[ref35] Bizmark N.; Schneider J.; Priestley R. D.; Datta S. S. Multiscale Dynamics of Colloidal Deposition and Erosion in Porous Media. Sci. Adv. 2020, 6, eabc2530 10.1126/sciadv.abc2530. 33188022 PMC7673751

[ref36] Erni-Cassola G.; Zadjelovic V.; Gibson M. I.; Christie-Oleza J. A. Distribution of Plastic Polymer Types in the Marine Environment; A Meta-Analysis. J. Hazard. Mater. 2019, 369, 691–698. 10.1016/j.jhazmat.2019.02.067. 30826562

[ref37] Gewert B.; Plassmann M. M.; MacLeod M. Pathways for Degradation of Plastic Polymers Floating in the Marine Environment. Environ. Sci. Process. Impacts 2015, 17, 1513–1521. 10.1039/c5em00207a. 26216708

[ref38] ASTM D5071-06 Standard Practice for Exposure of Photodegradable Plastics in Xenon Arc Apparatus. ASTM International 2021.

[ref39] Geosynthetics, T. UV Durability of TenCate Geosynthetics; Pendergrass: GA, 2019.

[ref40] Schneider C. A.; Rasband W. S.; Eliceiri K. W. NIH Image to ImageJ: 25 Years of Image Analysis. Nat. Methods 2012, 9, 671–675. 10.1038/nmeth.2089. 22930834 PMC5554542

[ref41] Meides N.; Menzel T.; Poetzschner B.; Löder M. G. J.; Mansfeld U.; Strohriegl P.; Altstaedt V.; Senker J. Reconstructing the Environmental Degradation of Polystyrene by Accelerated Weathering. Environ. Sci. Technol. 2021, 55, 7930–7938. 10.1021/acs.est.0c07718. 34018732

[ref42] Almond J.; Sugumaar P.; Wenzel M.; Hill G.; Wallis C. Determination of the Carbonyl Index of Polyethylene and Polypropylene Using Specified Area under Band Methodology with ATR-FTIR Spectroscopy. e-Polymers 2020, 20, 369–381. 10.1515/epoly-2020-0041.

[ref43] Hunter R. J. Foundations of Colloid Science/Robert J. Hunter; Oxford University Press: Oxford ; New York, 2001.

[ref44] Trozzolo A. M.; Winslow F. H. A Mechanism for the Oxidative Photodegradation of Polyethylene. Macromolecules 1968, 1, 98–100. 10.1021/ma60001a019.

[ref45] Ustunol I. B.; Gonzalez-Pech N. I.; Grassian V. H. PH-Dependent Adsorption of α-Amino Acids, Lysine, Glutamic Acid, Serine and Glycine, on TiO2 Nanoparticle Surfaces. J. Colloid Interface Sci. 2019, 554, 362–375. 10.1016/j.jcis.2019.06.086. 31306947

[ref46] Ma Y.; Wu Y.; Lee J. G.; He L.; Rother G.; Fameau A.-L.; Shelton W. A.; Bharti B. Adsorption of Fatty Acid Molecules on Amine-Functionalized Silica Nanoparticles: Surface Organization and Foam Stability. Langmuir 2020, 36, 3703–3712. 10.1021/acs.langmuir.0c00156. 32202121 PMC7311077

[ref47] Lee J. G.; Lannigan K.; Shelton W. A.; Meissner J.; Bharti B. Adsorption of Myoglobin and Corona Formation on Silica Nanoparticles. Langmuir 2020, 36, 14157–14165. 10.1021/acs.langmuir.0c01613. 33210541 PMC7735741

[ref48] Turner A.; Holmes L.; Thompson R. C.; Fisher A. S. Metals and Marine Microplastics: Adsorption from the Environment versus Addition during Manufacture, Exemplified with Lead. Water Res. 2020, 173, 115577 10.1016/j.watres.2020.115577. 32044597

[ref49] Woodall L. C.; Sanchez-Vidal A.; Canals M.; Paterson G. L. J.; Coppock R.; Sleight V.; Calafat A.; Rogers A. D.; Narayanaswamy B. E.; Thompson R. C. The Deep Sea Is a Major Sink for Microplastic Debris. R. Soc. Open Sci. 2021, 1, 140317 10.1098/rsos.140317. PMC4448771 26064573

[ref50] Kane I. A.; Clare M. A.; Miramontes E.; Wogelius R.; Rothwell J. J.; Garreau P.; Pohl F. Seafloor Microplastic Hotspots Controlled by Deep-Sea Circulation. Science 2020, 368, 1140–1145. 10.1126/science.aba5899. 32354839

[ref51] Barnes P. W.; Williamson C. E.; Lucas R. M.; Robinson S. A.; Madronich S.; Paul N. D.; Bornman J. F.; Bais A. F.; Sulzberger B.; Wilson S. R.; Andrady A. L.; McKenzie R. L.; Neale P. J.; Austin A. T.; Bernhard G. H.; Solomon K. R.; Neale R. E.; Young P. J.; Norval M.; Rhodes L. E.; Hylander S.; Rose K. C.; Longstreth J.; Aucamp P. J.; Ballaré C. L.; Cory R. M.; Flint S. D.; de Gruijl F. R.; Häder D.-P.; Heikkilä A. M.; Jansen M. A. K.; Pandey K. K.; Robson T. M.; Sinclair C. A.; Wängberg S.-Å.; Worrest R. C.; Yazar S.; Young A. R.; Zepp R. G. Ozone Depletion, Ultraviolet Radiation, Climate Change and Prospects for a Sustainable Future. Nat. Sustain. 2019, 2, 569–579. 10.1038/s41893-019-0314-2.

[ref52] Croxatto Vega G.; Gross A.; Birkved M. The Impacts of Plastic Products on Air Pollution - A Simulation Study for Advanced Life Cycle Inventories of Plastics Covering Secondary Microplastic Production. Sustain. Prod. Consum. 2021, 28, 848–865. 10.1016/j.spc.2021.07.008.

