# Supplementary material for: Effects of Weathering on Microplastic Dispersibility and Pollutant Uptake Capacity
Source: ACS Environ Au. 2022 Aug 31;2(6):549–55. doi: 10.1021/acsenvironau.2c00036 (PMC9673469; doi:10.1021/acsenvironau.2c00036)
Supplement: Supplementary file 1 — vg2c00036_si_001.pdf [file vg2c00036_si_001.pdf]

Effects of weathering on microplastic dispersibility and pollutant uptake capacity

Ahmed Al Harraq[1,†], Philip J. Brahana[1,†], Olivia Arcemont[1], Donghui Zhang[2], Kalliat T. Valsaraj[1], and Bhuvnesh Bharti[*,1]

[1]*Cain Department of Chemical Engineering, Louisiana State University, Baton Rouge, LA 70803, USA*
[2]*Department of Chemistry, Louisiana State University, Baton Rouge, LA 70803, USA*

*Corresponding author: bbharti@lsu.edu
†These authors contributed equally to the work

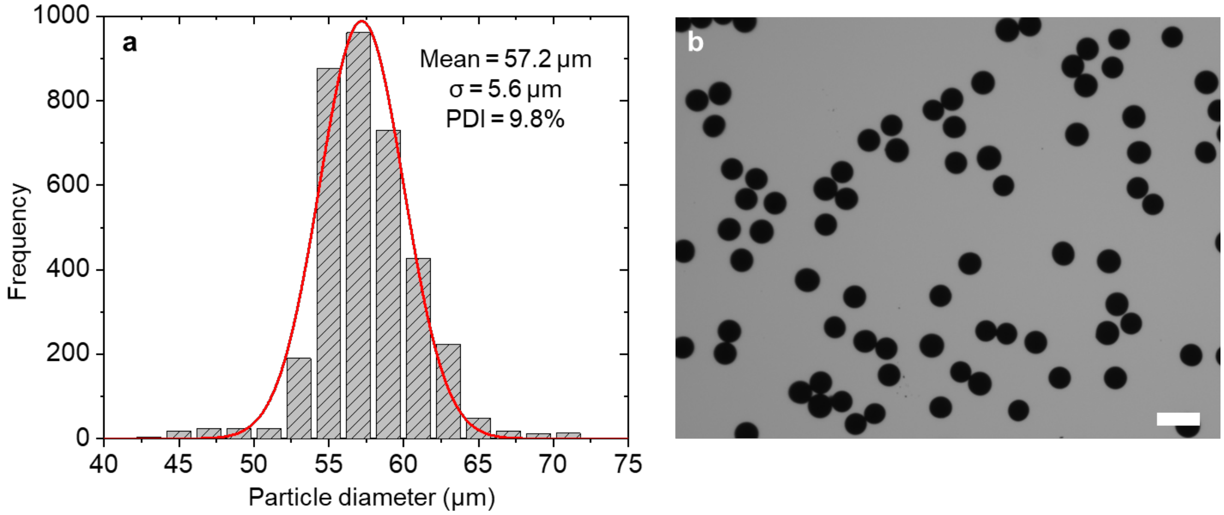

Fig. S1. a, Particle size distribution and **b,** optical microscopy image of polyethylene microplastics used. Here σ, is the standard deviation in the microplastic size as given by the log-normal distribution (red line) and PDI is the polydispersity index. Scale bar: 100 μm.

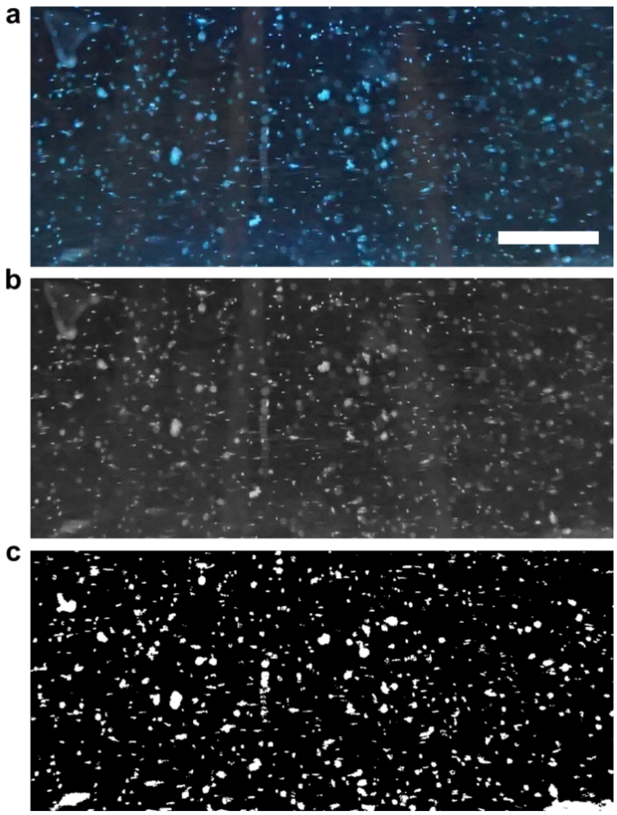

Fig. S2. The count of particles dispersed in the water bulk is done via image analysis (using ImageJ). **a**, An image of blue polyethylene microplastics is first rendered **b**, black and white before being **c**, binarized to obtain the approximate number of particles in a two-dimensional projection of the water volume. Scale bar: 0.5 cm.

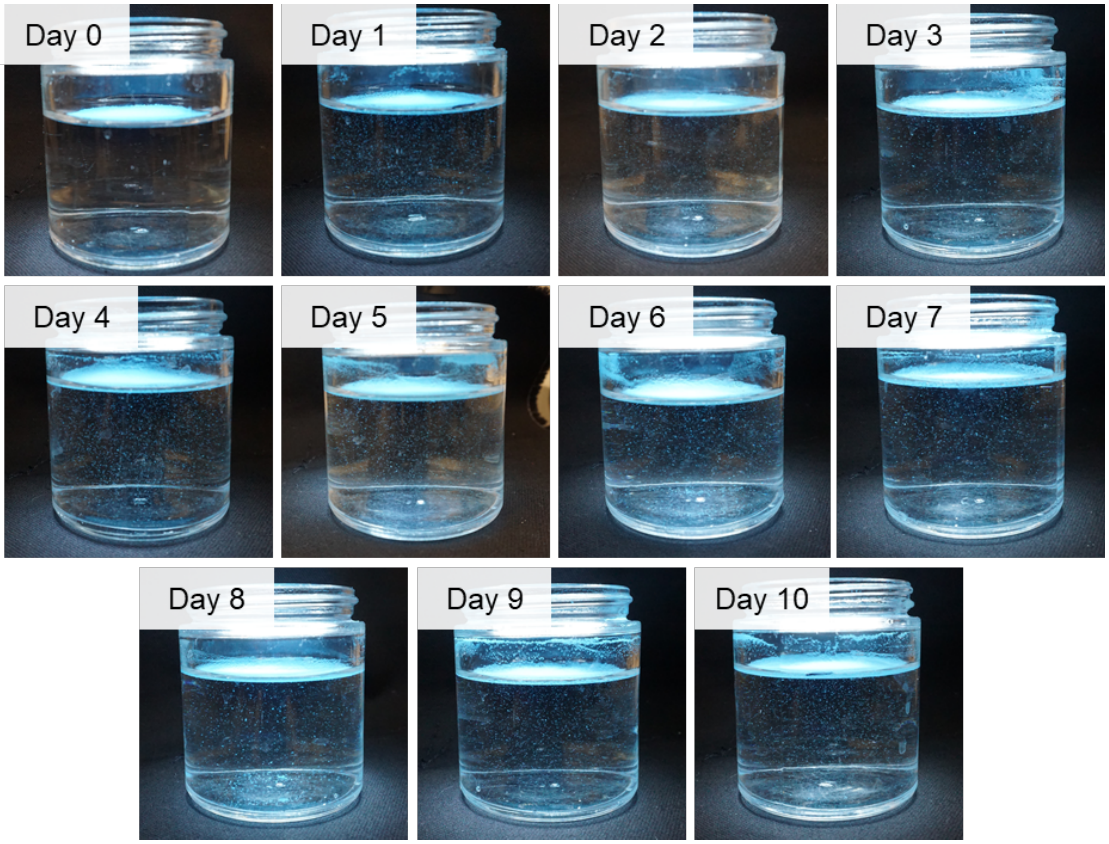

Fig. S3. Photographs of microplastics in water taken for each day (= 24 hrs) of weathering time.

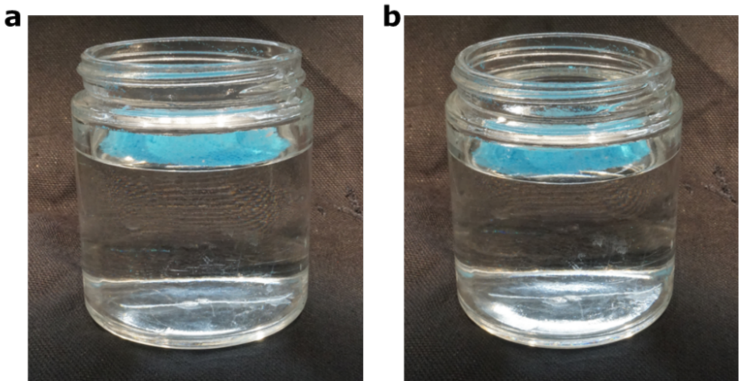

Fig. S4. Photographs of **a**, freshly prepared polyethylene microplastics in water and **b**, after keeping them in dark environment for 10 days, i.e., without weathering in the chamber. The microplastics show no change in dispersibility, unless they are weathered under the simulated sunlight.

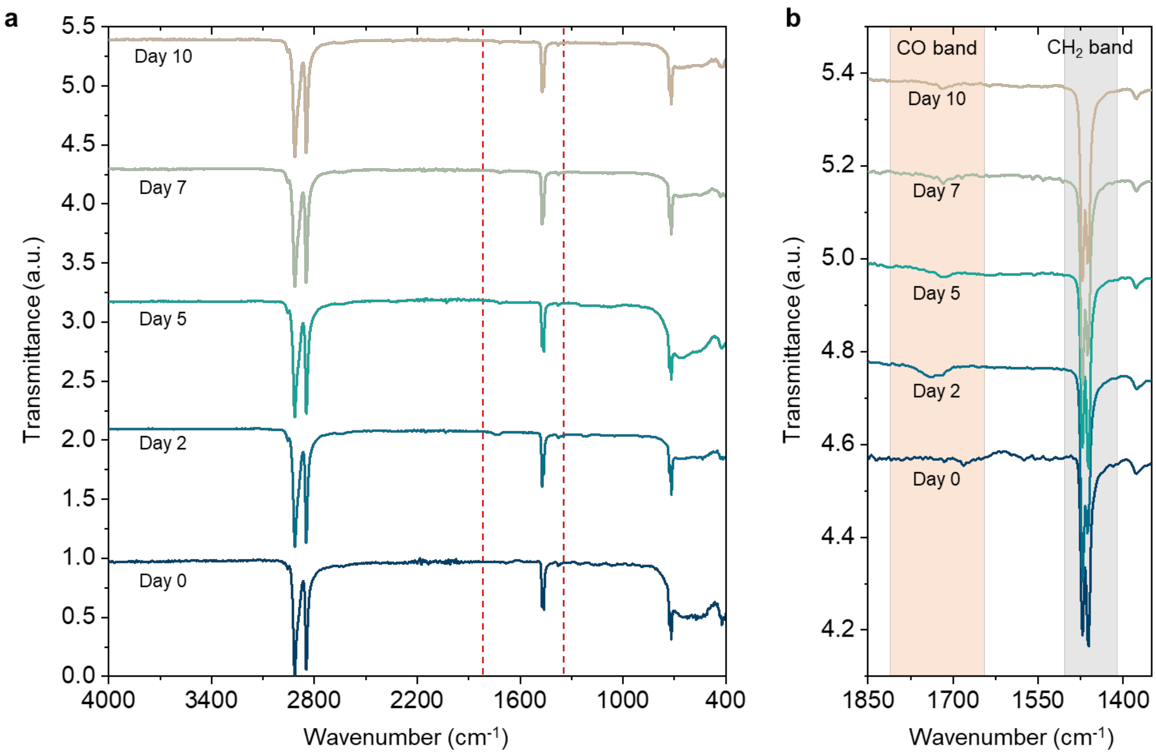

Fig. S5. **a**, Fourier-transform infrared (FTIR) spectra of polyethylene pellet samples with increasing weathering time from bottom to top. **b**, Highlight of the FTIR spectra near the carbonyl (CO) and methylene (CH_2) regions, showing the formation of a small peak in the 1650-1800 cm^{-1} band. The low intensity of CO peak relative to CH_2 peak indicates that only a small fraction of the surface groups are photooxidized during the weathering process.

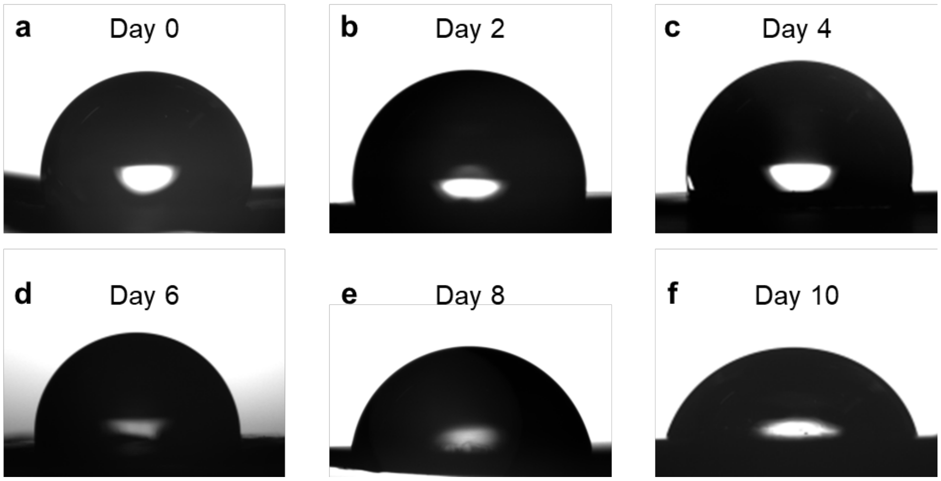

Fig. S6. Photographs of water droplet (2 μL) on pellets obtained from polyethylene microplastics and weathered for increasing number of days, showing a gradual decrease in the contact angle. The values of the water contact angle are provided in Figure 3 of the main article.

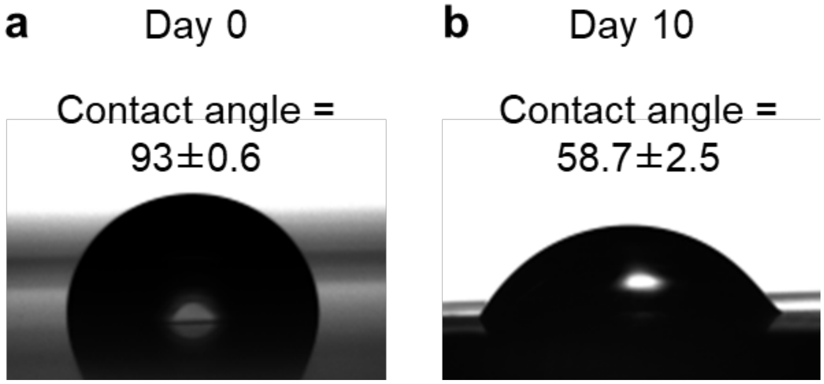

Fig. S7. Sample photograph of water droplets on polypropylene plates used to measure the water contact angle. The angle between the substrate and 2 μL water droplets decreases after weathering the plate for 10 days. The error is the standard deviation from measurements on 3 droplets.

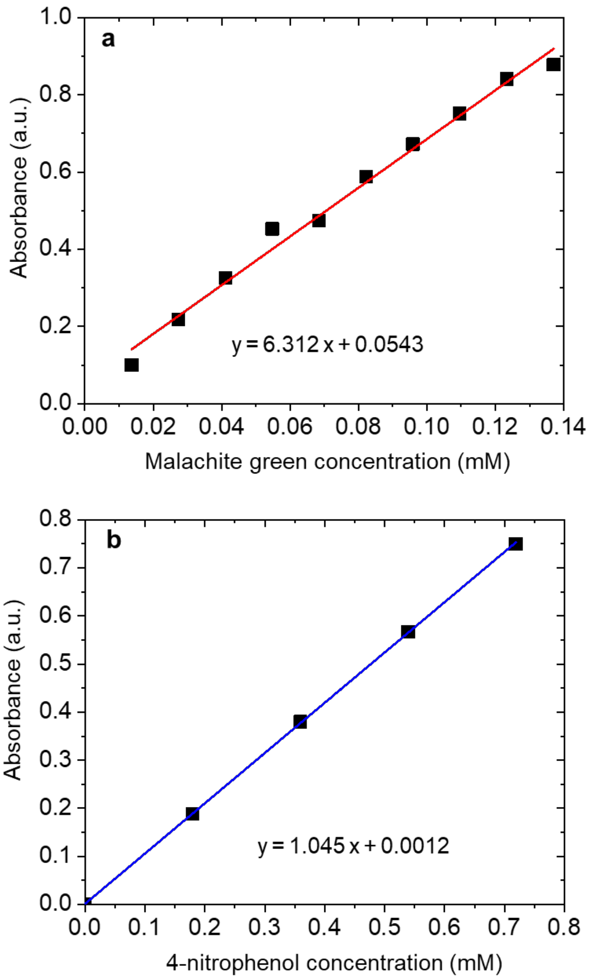

Fig. S8. Calibration curves of spectrophotometric absorbance with bulk concentration of **a**, malachite green and **b**, 4-nitrophenol in water. Black squares are experimentally determined values of absorbance at 618 nm for malachite green and 318 nm for 4-nitrophenol. The solid lines are linear fits to the data where the equation of the straight-line is provided in the respective plots.
